# Effects of Physiological Doses of Resveratrol and Quercetin on Glucose Metabolism in Primary Myotubes

**DOI:** 10.3390/ijms22031384

**Published:** 2021-01-30

**Authors:** Itziar Eseberri, Claire Laurens, Jonatan Miranda, Katie Louche, Arrate Lasa, Cedric Moro, Maria P. Portillo

**Affiliations:** 1 Nutrition and Obesity Group, Department of Nutrition and Food Science, Faculty of Pharmacy, University of Basque Country (UPV/EHU) and Lucio Lascaray Research Centre, 01006 Vitoria, Spain; itziar.eseberri@ehu.eus (I.E.); jonatan.miranda@ehu.eus (J.M.); arrate.lasa@ehu.eus (A.L.); 2 Bioaraba Health Research Institute, 01009 Vitoria, Spain; 3 CIBEROBN Physiopathology of Obesity and Nutrition, Institute of Health Carlos III (ISCIII), 28029 Madrid, Spain; 4 INSERM, UMR1048, Obesity Research Laboratory, Institute of Metabolic and Cardiovascular Diseases, 31432 Toulouse, France; claire.laurens@inserm.fr (C.L.); katie.louche@inserm.fr (K.L.)

**Keywords:** resveratrol, quercetin, glucose, human primary myotubes

## Abstract

Phenolic compounds have emerged in recent years as an option to face insulin resistance and diabetes. The central aim of this study was: (1) to demonstrate that physiological doses of resveratrol (RSV) or quercetin (Q) can influence glucose metabolism in human myotubes, (2) to establish whether AMP-activated protein kinase (AMPK) and protein kinase B –PKB- (Akt) pathways are involved in this effect. In addition, the effects of these polyphenols on mitochondrial biogenesis and fatty acid oxidation were analysed. Myotubes from healthy donors were cultured for 24 h with either 0.1 μM of RSV or with 10 μM of Q. Glucose metabolism, such as glycogen synthesis, glucose oxidation, and lactate production, were measured with D[U-^14^C]glucose. β-oxidation using [1–^14^C]palmitate as well as the expression of key metabolic genes and proteins by Real Time PCR and Western blot were also assessed. Although RSV and Q increased pgc1α expression, they did not significantly change either glucose oxidation or β-oxidation. Q increased AMPK, insulin receptor substrate 1 (IRS-1), and AS160 phosphorylation in basal conditions and glycogen synthase kinase 3 (GSK3β) in insulin-stimulated conditions. RSV tended to increase the phosphorylation rates of AMPK and GSK3β. Both of the polyphenols increased insulin-stimulated glycogen synthesis and reduced lactate production in human myotubes. Thus, physiological doses of RSV or Q may exhibit anti-diabetic actions in human myotubes.

## 1. Introduction

Insulin resistance and diabetes currently represent pandemic diseases at a global level. In 2016, diabetes caused an estimated 1.6 million deaths, and high blood glucose levels were responsible for 2.2 million deaths [1]. For this reason, great effort is being made to find new strategies in the fight against this disease.

Skeletal muscle is the main tissue that is involved in glycaemic control in the post-prandial state. It contributes to 85% of the whole body glucose uptake, which is essential in avoiding insulin resistance development [2]. Furthermore, it should be noted that 80% of glycogen storage is located in the skeletal muscle. Glucose transporter 4 (GLUT4) mediates glucose uptake, where the activation can be promoted in an insulin-dependent or insulin-independent manner [3]. In the former case, when insulin binds its receptor, the latter phosphorylates insulin receptor substrate 1 (IRS-1) and new binding sites for other proteins are originated. Some of these proteins belong to the phosphoinositide 3-kinases (PI3K) family, which stimulate Akt through the PI3K pathway. This fact leads not only to GLUT4 glucose transporter activation, but also to glycogen synthase kinase 3 (GSK3β) inhibition and, thus, to glycogen synthase (GS) activation [2,4,5]. AMP-activated protein kinase (AMPK) is another GLUT4 activator, although in an insulin-independent manner [6]. AMPK is also considered to be a critical regulator of lipid oxidation [7], which regulates exercise-related metabolic adaptations. Consequently, it can be a therapeutic target for several metabolic disorders, including obesity and diabetes [6].

Other stimuli, such as drugs, energy restriction, or dietary compounds, can trigger glucose uptake through both insulin-dependent and insulin-independent pathways, as occurs with physical activity in AMPK activation [8,9,10]. Among these stimuli, phenolic compounds have emerged as an alternative in recent years. Several in vitro and in vivo studies have shown that resveratrol (RSV) and quercetin (Q) have anti-diabetic properties [11,12,13]. Although both of the phenolic compounds are multi-target molecules, it seems that skeletal muscle is involved in achieving this effect. A clear example of this is the large amount of research that has been conducted to date with RSV and Q in muscle tissue or muscle cells, mainly in rodent models [14,15]. Nevertheless, the extrapolation of these results to humans is limited, in that the doses that are used to analyse their effect in in vitro and in vivo studies are not always in the concentration that these compounds reach in human tissues and cells after oral intake [16,17,18,19]. For this reason, in the present study we analyse whether doses of RSV or Q in the range of the amounts that are found in tissues after oral treatments with these phenolic compounds are able to influence glucose metabolism in human myotubes. In addition, we determine whether AMPK and Akt pathways are involved in this effect. To conclude, the effect of these polyphenols on mitochondrial biogenesis and fatty acid oxidation is also assessed.

## 2. Results

### 2.1. Results in Cytotoxicity Assay of RSV and Q

The potential cytotoxic effect of both polyphenols was analysed using a commercial kit, and no statistical change in the release of adenylate kinase (AK) to the incubation media between the treated and control cells was observed. The obtained data were, as follows: 2332.31 ± 403.3 RLU/mg protein in the control group, 2876.12 ± 208.98 RLU/mg protein in RSV group, and 2307.66 ± 220.18 RLU/mg protein in Q group.

### 2.2. Effects of RSV and Q in the Expression of Mitochondrial and Cytosolic Genes

**Figure 1:**
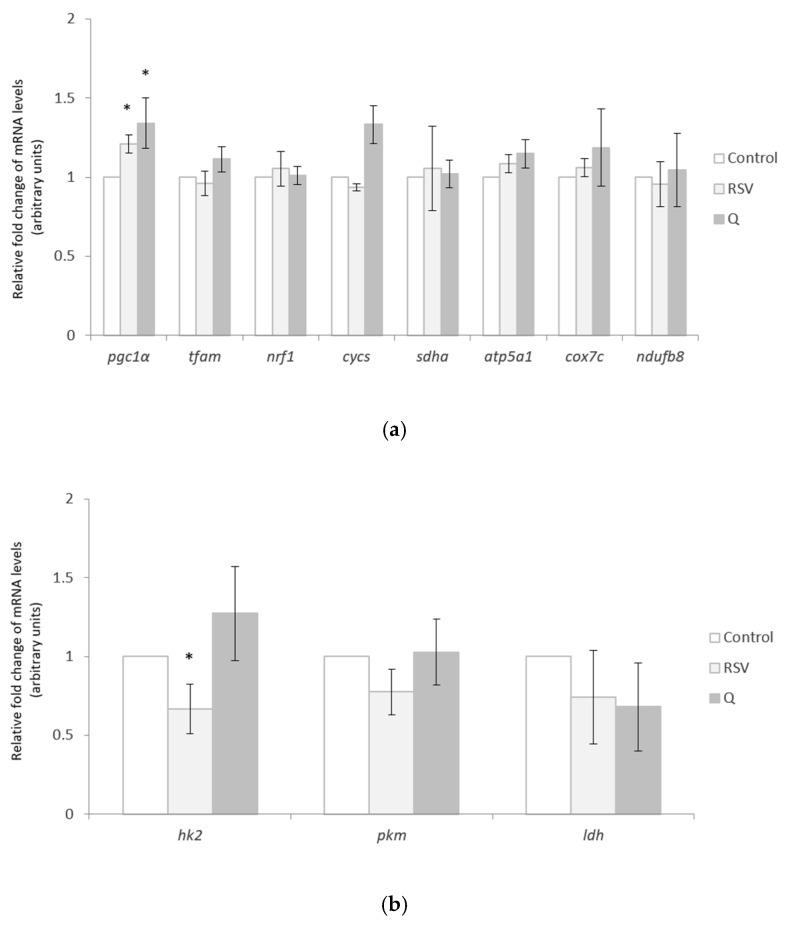
Mitochondrial (**a**) and cytosolic (**b**) gene expression in human myotubes treated with 0.1 µM of resveratrol (RSV) and 10 µM of quercetin (Q) for 24 h. All of the data are presented as the mean ± SEM of two independent experiments (*n* = 6 per group). Comparisons between biological replicates of each treatment group and biological replicates of the control group were analysed by Student’s *t*-test. The asterisks represent differences versus the controls (* *p* < 0.05).

The expression of *peroxisome proliferator-activated receptor gamma coactivator 1-alpha* (*pgc-1α*), *mitochondrial transcription factor A (tfam)*, and *nuclear respiratory factor 1 (nrf1)*, genes that regulate mitochondrial biogenesis, and that of *cytochrome C (cycs)*, *succinate dehydrogenase complex, subunit alpha (sdha), ATP synthase, H+ transporting, mitochondrial F1 complex, alpha subunit 1 (atp5a1), cytochrome c oxidase subunit7C (cox7c)*, and *NADH dehydrogenase (ubiquinone) 1 beta subcomplex 8 (ndufb8)*, which encode the respiratory chain components, was measured. Among them, only *pgc1α* expression increased after cell treatment with either RSV or Q (Figure 1a). The mRNA levels of *hexokinase 2 (hk2), pyruvate kinase M1/2 (pkm)*, and *lactate dehydrogenase A (ldh)*, cytosolic genes related to glucose oxidation and lactate production were also measured and the results yielded a reduction of *hk2* expression after RSV treatment (Figure 1b).

### 2.3. Effects of RSV and Q on Glucose Uptake and Oxidation and Palmitate Oxidation

**Figure 2:**
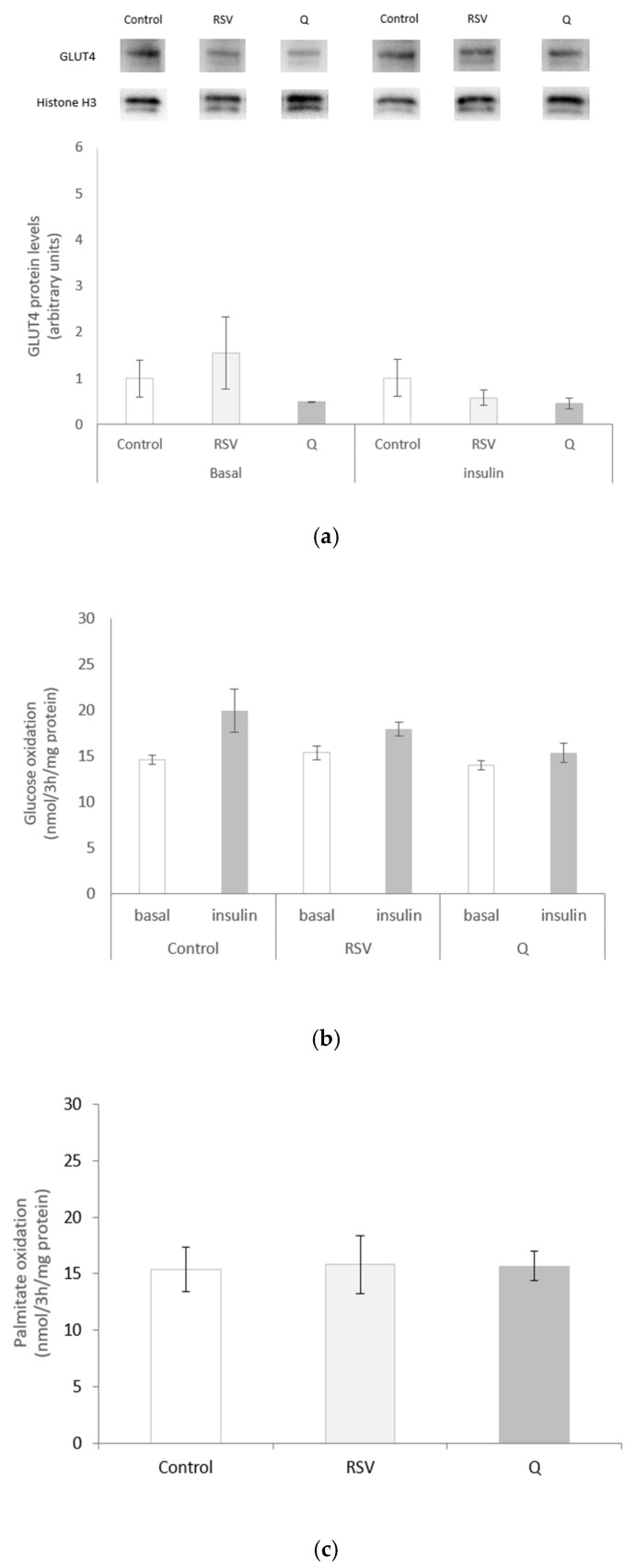
GLUT4 protein expression (**a**), glucose oxidation (**b**) and palmitate oxidation (**c**) in human myotubes treated with 0.1 µM of resveratrol (RSV) and 10 µM of quercetin (Q) for 24 h. All of the data are presented as the mean ± SEM of six (**a**) or three (**b**,**c**) biological replicates. Comparisons between each treatment group and the control group were analysed by Student’s *t*-test.

Neither RSV nor Q modified GLUT4 protein expression (Figure 2a). The cells were treated with both molecules under basal and insulin-stimulated conditions, and labelled glucose was added to the media, in order to test whether RSV or Q modified glucose oxidation. After CO_2_ quantification, it can be observed that insulin-treated cells were not statistically modified as compared to the basal state (C basal vs. C insulin *p* = 0.09; RSV basal vs. RSV insulin *p* = 0.076; Q basal vs. Q insulin *p* = 0.319). Furthermore, the result showed that RSV or Q treatments did not modify the flow of glucose to oxidation in the basal (C vs. RSV *p* = 0.443; C vs. Q *p* = 0.483) or in the stimulated state (C vs. RSV *p* = 0.463; C vs. Q *p* = 0.149) (Figure 2b). In addition, we wanted to analyse whether cell treatment could enhance fatty acid β-oxidation, so labelled palmitate was added to incubation media and the resulting CO_2_ was quantified. No changes were observed between the control and cells treated with each polyphenol (Figure 2c).

### 2.4. Effects of RSV and Q in Glucose Homeostasis

**Figure 3:**
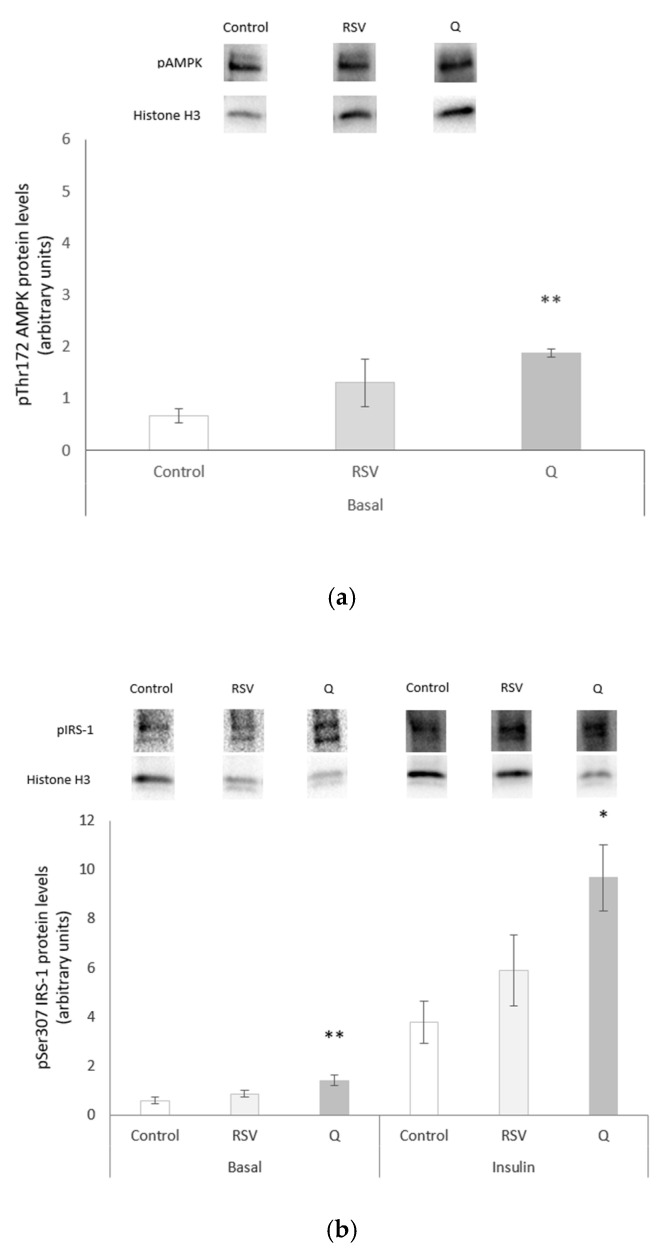

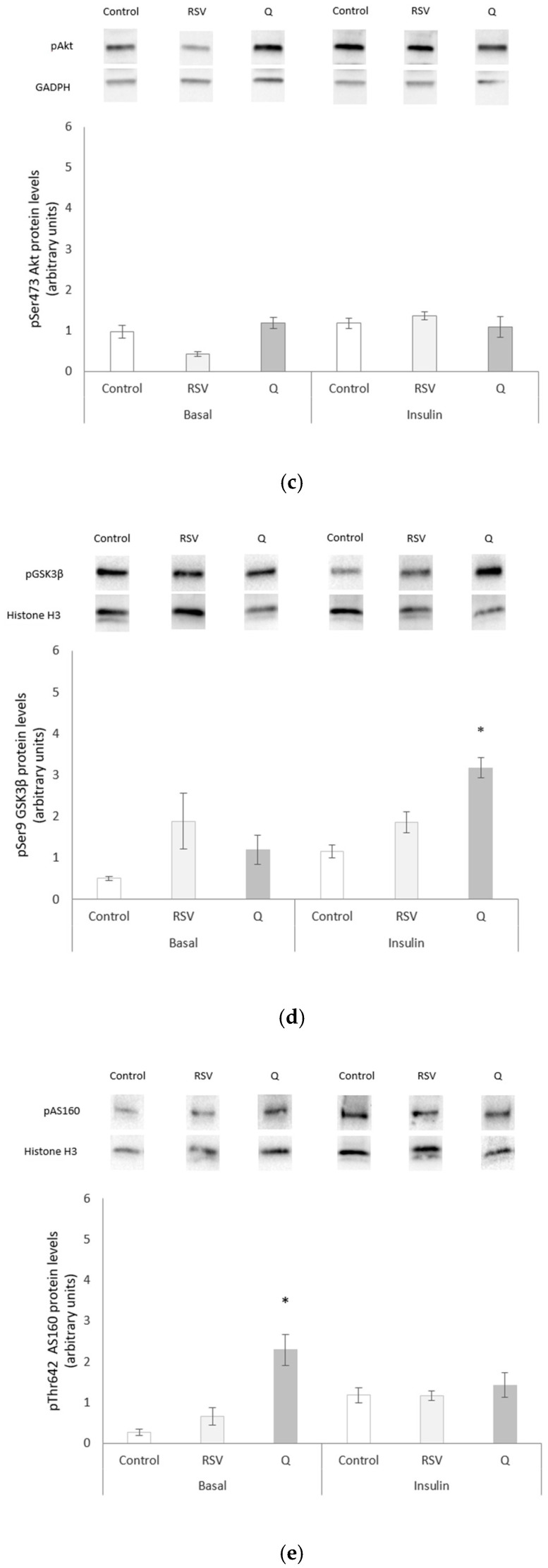
Protein expression of AMPK (**a**), IRS-1 (**b**), Akt (**c**), GSK3β (**d**), and AS160 (**e**) in human myotubes that were treated with 0.1 µM of resveratrol (RSV) and 10 µM of quercetin (Q) for 24 h in the presence or absence of insulin, with the exception of AMPK, which was measured only in basal conditions. Target protein bands are shown on the chart top as representative blot images. All of the data are presented as the mean ± SEM of six biological replicates. Comparisons between each treatment group and the control group were analysed by Student’s *t*-test. The asterisks represent differences versus the controls (* *p* < 0.05; ** *p* < 0.01).

The phosphorylated protein expression of AMPK, IRS-1, protein kinase B –PKB- (Akt), and AKT Substrate of 160 kDa (AS160) was measured to test the effect of RSV and Q on the insulin-signalling cascade. The expression of GSK3β, a key enzyme that is involved in glycogen synthesis, was also assessed. This analysis was carried out under basal and insulin-stimulated conditions, with the exception of AMPK, which is insulin-independently regulated. Under basal conditions, Q increased the phosphorylation of AMPK, IRS-1, and AS160 (Figure 3a,b,e), while increasing IRS-1 and GSK3β expression under insulin-stimulated conditions (Figure 3b,d). Although RSV notably increased the phosphorylation of some proteins, such as AMPK (197%) and GSK3β (161%), these enhancements did not reach statistical significance (Figure 3a,d).

### 2.5. The Effect of RSV and Q in Glycogen Synthesis and Lactate Production

**Figure 4:**
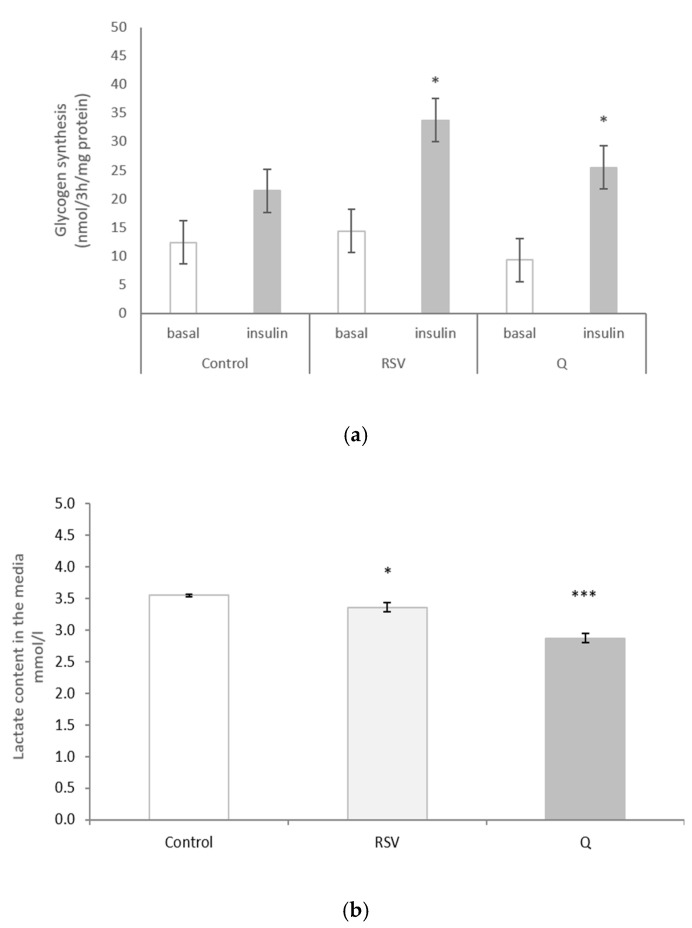
Glycogen synthesis (**a**) and lactate content in the media (**b**) in human myotubes treated with 0.1 µM of resveratrol (RSV) and 10 µM of quercetin (Q) for 24 h. Glycogen synthesis data are presented as the mean ± SEM of three biological replicates. The data of lactate content in the media are presented as the mean ± SEM of six biological replicates. Comparisons between each treatment group and the control group were analysed by Student’s *t*-test. The asterisks represent differences versus the controls (* *p* < 0.05; (*** *p* < 0.001)).

The effect of RSV and Q on glycogen synthesis was tested under basal and insulin-stimulated conditions. We observed that both of the polyphenols significantly increased insulin-stimulated glycogen synthesis (Figure 4a). The increase in insulin-induced glycogen synthesis *versus* the increase under basal conditions was calculated. Both phenolic compounds increased this ratio when compared to the controls (*p* < 0.05). Regarding lactate production, after cell incubation with 0.1 µM of RSV and 10 µM of Q, the content that was present in the incubation media was significantly lower than that present in the control cells (Figure 4b).

## 3. Discussion

Several research studies that have been conducted in the last decades have demonstrated that phenolic compounds decrease the incidence of cardiovascular diseases, several types of cancer, liver disorders, obesity, and type 2 diabetes, among others [20,21]. In the case of RSV or Q, the anti-diabetic effect emerges as one of the most studied positive effects in experiments that were conducted in cell cultures and animal models. In the same way, this effect has been observed in clinical trials, where the used doses range widely between 10 mg/day and 3 g/day in the case of RSV and 30mg/day to 1 g/day in the case of Q [22,23,24]. It is important to bear in mind that these studies have been performed using doses that can be achieved only by phenolic compound supplementation. Actually, the average dietary intake of both RSV and Q is much lower; regarding RSV, Zamora-Ros estimated that stilbene intake was around 2–3 mg per day, with total RSV intake being almost 1 mg per day [25]. By contrast, Q intake is clearly higher than that of RSV, its mean dietary ingestion has been estimated to be between 5 and 40 mg per day, although significantly higher amounts have been reported in individuals with a very high consumption of fruit and vegetables [26]. Concerning their safety, RSV and Q have been reported to be safe in humans when administered at doses up to 5 g/day and 1g/day respectively [27,28].

However, the specific contribution of skeletal muscle in the described beneficial effects for both molecules in humans has not been clearly elucidated, mainly due to the difficulty in obtaining human skeletal muscle samples, as the lack of references in reviews demonstrates [22,29,30,31]. Conversely, the doses used in the majority of the reported studies are far from those found in human plasma and tissues after oral administration of both compounds, as stated in the Introduction section. Overall, RSV and Q are found in nanomolar and low micromolar range, suggesting that doses as high as 50, 100 or 200 µM, very commonly used in in vitro studies with incubated cells, are hardly found [32,33,34,35,36,37]. For this reason, in the present study, we wanted to provide more information regarding the potential activity of both molecules in human muscle, by using healthy human myotubes and doses of RSV and Q that are closer to those that were achieved in plasma and tissues.

With this intention, the first challenge that we encountered was selecting the doses for cell treatment. In a study that was conducted by Brown et al., repetitive administration of RSV at doses ranging from 0.5 g to 5 g per day resulted in plasma concentrations of the phenolic compound of around 4 µM [38]. In a clinical trial that was carried out by Olthof et al., the ingestion of 150 mg of Q glycosides led to a Q plasma concentration of around 5 µM [19]. In view of this information, we decided to choose doses that ranged from 0.1 to 10 µM as potential active doses. *Pgc1α* was chosen in order to verify whether selected doses of RSV and Q were able to exert physiological effects on cells; this gene is the master regulator of fatty acid oxidation and mitochondrial biogenesis, and it has been demonstrated to be affected by both phenolic compounds [19,39,40,41,42]. In the case of RSV, incubation with 0.1 µM for 24 h was enough to induce a significant increase in *pgc1α* expression, whereas, in the case of Q, 10 µM was the lowest active dose (data not shown). It is important to highlight that none of the treatments had any toxic effects on myotubes. These effects are in good accordance with those that were obtained in other studies while using RSV at 25 µM for 72 h or Q 50 µM for 18 h in C2C12 cells [43,44] or RSV at 100 µM for 4 h in human primary muscle cells [45].

The activation of insulin-dependent pathway—Akt—or the stimulation of an insulin-independent pathway, induce glucose uptake, with AMPK as the key promoter. Because exercise stimulates AMPK activity in muscle, and RSV has been demonstrated to activate molecular mechanisms analogous to exercise training, it might be inferred that it also probably stimulates this insulin independent pathway [39]. In the present study, no changes in the phosphorylation of IRS-1, Akt, AS160, and AMPK were observed in cells that were treated with RSV under basal conditions, which suggested that this polyphenol did not act directly on the proteins that are involved in the insulin-signalling cascade or the insulin-independent pathway mediated by AMPK. These results are in good accordance with the observed lack of effect on glucose uptake and oxidation, as well as on glycogen synthesis. The same situation took place under insulin stimulation. The present results suggest that, under our experimental conditions, a dose of RSV in the physiological range do not promote glucose utilization.

We revised the literature in order to compare our results with those reported by other authors and we found controversial results. Breen et al. showed that a minimum dose of 25 µM RSV and 30 min of treatment were needed to induce a significant glucose uptake enhancement in L6 myotubes [46]. However, in another study using the same cell line, lower doses of RSV (1 µM) were able to increase the glucose uptake by both insulin and AMPK signalling [47]. On the other hand, Skrobuk and co-workers found that RSV at 1 or 10 µM for 4h in primary muscle cells increased basal glucose uptake, whereas exposure to very high concentrations (100 and 200 µM) led to a decrease [45]. Fröjdö et al. observed a similar phenomenon, when human primary cells were treated with doses ranging from 5 to 100 µM [48]. In the same line, Kaminski et al., showed decreased *glut4* mRNA levels after 30 µM RSV treatment for 48 h in C2C12 myotubes [49]. The discrepancies between our results and those that were reported by other authors, as well as among the published studies, can be due to RSV dosage [45]. It is important to emphasize that the dose used in the present study (0.1 µM) was lower than those that were used in all of the reported studies. In addition, other factors that are related to the experimental design that can explain these differences, because they have been described as important influencing factors for the effect of RSV on glucose uptake, are the treatment period length or the cell origin. Thus, Barger et al. observed that, while RSV enhanced insulin-stimulated glucose transport in the soleus muscle, it did not in extensor digitalis muscle of mice, which means that the type of muscle utilised is also relevant [50].

Concerning Q, although this polyphenol was able to increase IRS-1 phosphorylation in the basal state, no changes were observed in the downstream proteins of the insulin-signalling cascade. Because AMPK phosphorylation was enhanced, it can be proposed that this enzyme was responsible for the increase in AS-160 phosphorylation. However, it seems that AS-160 boost was not enough for rising glucose uptake in cells due to the lack of change observed in GLUT4 protein content. This fact is in good accordance with no variation noted in glucose oxidation and glycogen synthesis after Q treatment.

Finally, Q did not potentiate the effect of insulin on the phosphorylation of proteins that are involved in insulin signalling cascade, with the exception of IRS-1. The attenuation of the PI3K/AKT pathway by Q could be proposed as a potential explanation to justify this result; it has been described that Q increases the expression of PTEN, a PI3K/AKT pathway natural inhibitor phosphatase and tensin homolog, in cancer cells [51]. Other polyphenols, such as curcumin and xanthohumol, also increase the expression of PTEN in glioblastoma cells and myocytes, respectively [52,53,54]. Of note, such a lack of correlation between the phosphorylation of Akt and AS-160 is not without precedent. In a previous study from our group, which was devoted to comparing the effects of RSV and energy restriction on insulin signalling cascade in skeletal muscle, increased phosphorylation of AS-160 was not accompanied by increases in the phosphorylation status of IRS-1 and Akt [55]. Consequently, this issue needs further research.

By comparing our results with the literature, we observed that they are in line with those that were observed in other studies, where the incubation of L6 or C2C12 murine cells with 25 and 50 µM of Q for 18 or six hours promoted an increase in AMPK phosphorylation, but not through Akt activation [44,56]. By contrast, Jiang et al. observed a clear activation of Akt in a study that was carried out in L6 rat muscle cells by using very low doses of Q (0.1 and 10 nM) [8]. Dai et al. demonstrated that, after 24 h treatment in C2C12 myotubes, the dose of 5 and 10 µM of Q were without effect, whereas the dose of 20 µM induced Akt activation [57]. Hence, as in the case of RSV, the dose of Q is a crucial factor in for the effectiveness of this phenolic compound.

It is well known that skeletal muscle is an important storage tissue for glycogen, as it is the main site for glucose disposal in humans. However, glycogen synthesis is compromised in insulin-resistant or type 2 diabetes patients, which could be caused by GS dysfunction or by a lack of glucose to be stored as glycogen, which, in turn, reduces GS activity [58]. In fact, it is not totally established which comes first, glucose supply or a reduction in GS activity, although the first option seems to be more likely, since insulin-resistant subjects had normal glycogen synthesis rates after exercise training [58,59]. Bearing this in mind, promising results were obtained for RSV or Q in the present study, because both of the phenolic compounds were able to enhance glycogen synthesis and decrease the lactic acid content in human muscle cells, when incubated in the presence of insulin. These results are not in good accordance with those that were reported by Skrobuk et al., who observed a reduction in glycogen content in human primary myocytes after 100 µM RSV treatment for 4h [45]. By contrast, in the case of Q, the results concerning lactic acid in stimulated C2C12 cells presented in the patent entitled “methods for reducing lactate concentration” are similar to those that are found in the present study [60].

As previously described in this Discussion section, although RSV or Q treatments increased the mRNA levels of pgc1α, the expression of other genes that regulate mitochondrial biogenesis and genes of the respiratory chain were not modified. These results, together with the lack of effect on palmitate and glucose oxidation, support the hypothesis that the number of mitochondria was not altered after RSV or Q treatments in our experiment using human muscle cells. In contrast with our results, Skrobuk et al. reported that the incubation of primary human muscle cells with 100 µM of RSV for 4 h, a dose clearly higher than that used in the present study, was able to decrease palmitate oxidation without modification of PGC1α acetylation, and that incubation with RSV at 1 µM for 4h increased PGC1α acetylation [45]. Altogether, these results reinforce the importance of the dose when assessing the effects of RSV. With regard to clinical studies that were conducted using RSV, while some of them confirmed the mitochondriogenic effect that was observed in animals [18,61,62], others did not reveal any effect in muscle mitochondria after RSV intervention [63,64,65]. In the case of Q, Nieman et al. [66] did not show any effect on mitochondrial biogenesis in thirty-nine trained cyclists after a two-week treatment with 1 g/day of Q and three days of heavy exertion. Nevertheless, one year later, the same authors described Q influence on moderate exercise performance and muscle mitochondrial biogenesis in physically sedentary young patients who received 1 g/day of Q for two weeks [67]. Thus, both Nieman’s trial results showed that different conditions, such as exercise, age, previous metabolic state, dose, and treatment period length, can have a relevant influence on the effect of Q on mitochondrial biogenesis in muscle.

It is well known that polyphenols show low bioavailability [68,69,70]. Indeed, after oral ingestion of phenolic compounds, these undergo intense phase II metabolism in both the intestine and liver. Those phase II reactions include glucuronidation, sulfatation, and methylation, catalysed by uridine-5´-diphosphate glucuronosyltransferases (UGT), sulfotransferases (SULT) and catechol-O-methyltransferases (COMT), respectively [71]. This explains why their metabolites are found in plasma and tissues, even at concentrations that are higher than those of their parent compounds [71,72]. For this reason, it is crucial to study, in vitro, the effects of the main metabolites of RSV and Q, under the belief that they can contribute to the observed beneficial effects. Nevertheless, the activity of the parent compounds should also be measured, as we did in the present study; if they are active, the amounts that are found in target tissues may be responsible for a part of the effect that was observed after its in vivo administration, as we demonstrated in previous studies. In these studies, we found that the delipidating effect of RSV in 3T3-L1 maturing adipocytes was due to the effects of the parent compound and two of its main metabolites, trans-resveratrol-4-O-glucuronide and trans-resveratrol-3-O-sulfate, as well as the effects on mature adipocytes were due to RSV and to trans-resveratrol-3-O-glucuronide and trans-resveratrol-4′-O-glucuronide [73]. With regard to Q metabolites, we observed that the parent compound and the metabolite quercetin-3-O-glucuronide were both responsible for the anti-adipogenic effect that was induced in 3T3-L1 pre-adipocytes [74]. Finally, it should be pointed out that the deconjugation of phenolic compounds in tissues can occur, thus increasing the actual amount of the parent compounds [75,76,77].

## 4. Materials and Methods

### 4.1. Experimental Design

Satellite cells from rectus abdominis of healthy male subjects (age 34.3 ± 2.5 years, BMI 26.0 ± 1.4 kg/m^2^, fasting glucose 5.0 ± 0.2 mM) were obtained after abdominal surgery interventions and kindly provided by Prof. Arild C. Rustan (Oslo University, Oslo, Norway). Informed written consent was obtained from all of the participants and the ethical aspects were considered as previously described [78]. The ethical approval number was 2013-A01543-42. The cells were grown in Dulbecco’s modified Eagle’s medium (DMEM) low glucose-Glutamax^TM^ (GIBCO, BRL Life Technologies, Grand Island, NY, USA) supplemented with 10% Foetal Bovine Serum (FBS) (GIBCO, BRL Life Technologies, Grand Island, NY, USA) and growth factors. At 90% of confluence, myoblasts were differentiated from myotubes by switching to α-minimum essential medium (α-MEM) low glucose-Glutamax^TM^ (GIBCO, BRL Life Technologies, Grand Island, NY, USA), 2% FBS, and fetuin (0.5 mg/mL) (Sigma-Aldrich Corporation, St. Louis, MO, USA) until myoblasts were harvested five days after the induction of differentiation. Cells were maintained at 37 °C in a humidified 5% CO_2_ atmosphere and both incubation media were changed every two days.

### 4.2. Cell Treatment

Myotubes were incubated with either 0.1 μM of RSV or 10 μM of Q (Sigma, St. Louis, MO, USA), diluted in 95% ethanol for 24 h, and, afterwards, cells were harvested. In the case of the control group the same volume of the vehicle (ethanol 95%) was used.

### 4.3. Cytotoxicity Assay

Cytotoxicity assay was carried out using the ToxiLight^TM^ bioassay kit (Lonza, Walkersville, MD, USA) following the manufacturer´s instructions. The activity of released AK from damaged cells was c measured by means of chemiluminescence.

### 4.4. Glycogen Synthesis Assay

Myotubes were pre-incubated in a glucose and serum-free medium for 90 min in order to obtain metabolically consistent cells, followed by 3 h incubation using DMEM supplemented with D[U-^14^C]glucose (1μCi/mL) with or without 100 mM of insulin. Following incubation, the cells were solubilised in KOH 30% and glycogen synthesis was determined, as previously described [79]. The total glycogen content was spectrophotometrically measured after complete hydrolysis into glucose by α-amiloglucosidase. All of the assays were performed in duplicate and data were normalized to cell protein content.

### 4.5. Glucose Oxidation Assay

Myotubes were pre-incubated in a glucose and serum-free medium for 90 min, as in the case of glycogen synthesis assay. In order to study basal and insulin-mediated glucose oxidation, cells were incubated with DMEM supplemented with D[U-^14^C]glucose (1 μCi/mL) in the presence or absence of 100 mM of insulin. Following incubation, glucose oxidation was determined by counting the ^14^CO_2_ released into the culture medium. All of the assays were performed in duplicate, and the data were normalized to cell protein content, as previously described [80].

### 4.6. Palmitate Oxidation Assay

Myotubes were pre-incubated for three hours with [1–^14^C]palmitate (1 µCi/mL; PerkinElmer, Boston, Massachusetts) and non-labelled (cold) palmitate, without glucose. Palmitate was coupled to a fatty acid (FA)–free BSA in a molar ratio of 5:1. After incubation, ^14^CO_2_ and ^14^C-ASM were measured, as previously described [80]. All of the assays were performed in duplicate and data were normalized to cell protein content.

### 4.7. Measurement of Lactate Content in the Media

After cell treatment, aliquots of the incubation media were removed and analysed for lactate quantification using a commercial kit and following the manufacturer´s instructions. The results were expressed as mmol/L.

### 4.8. RNA Preparation and Quantitative Real Time PCR

The RNA samples from treated cells were extracted while using RNeasy mini kit (QIAGEN, Valencia, California, USA) following the manufacturer´s instructions. The integrity of the RNA extracted from all of the samples was verified and quantified using a NanoDrop ND-1000 spectrophotometer (Thermo Scientific, Wilmington, DE, USA). 1 μg of total RNA of each sample was reverse-transcribed to first-strand complementary DNA (cDNA) using the MultiScribe reverse transcriptase method (Applied Biosystems, Foster City, CA, USA) on a GeneAmpPCRSystem 9700 (Applied Biosystems, Foster City, CA, USA). Relative *pgc-1α*, *tfam*, (*nrf1*, *cycs*, *sdha*, *atp5a1*, *cox7c*, and *ndufb8* were quantified using Real-time PCR with a StepOne-Plus real-time PCR system (Applied Biosystems, Foster City, CA, USA). *Hk2*, *pkm* and *ldha* were measured by TaqMan^®^ Gene Expression Assays (Hs00606086_m1, Hs00761782_s1 and Hs01378790_g1 respectively) in MyiQ™ Single-Color Real-Time PCR Detection System (BioRad, Hercules, CA, USA). The *Rplp0* mRNA levels were similarly measured and served as the reference gene. The amplification reaction was performed in duplicate on 0.67 µL of cDNA and the amplification parameters were, as follows: 50 °C for two minutes, 95 °C for 10 min, 40 cycles of 95 °C for 15 s, and 60 °C for one minute. All fo the sample mRNA levels were normalized to Rplp0 values and the data were expressed as relative fold changes of threshold cycle (Ct) value relative to controls using the 2^−ΔΔCt^ method [81].

### 4.9. Western Blot Analysis

The myotubes were incubated for 20 min in DMEM-low glucose-Glutamax in the presence or absence of 100 nM insulin. Afterwards, the cells were harvested in a RIPA buffer (Sigma, St. Louis, MO, USA) complemented with 10 µL/mL protease inhibitor, 10 µL/mL phosphatase I inhibitor and 10 µL/mL phosphatase II inhibitor. Afterwards, the protein concentration was determined by BCA reagent (Thermo Scientific, Rockfold, IL, USA). 20 µg of total protein were run on 4–15% Mini-PROTEAN^®^ TGX™ Precast Gels (Bio-Rad, Hercules, CA, USA), electroblotted onto PVDF membranes (Millipore, Bradford, MA, USA), and immunodetected with ChemiDoc MP imaging system (BioRad, Hercules, CA, USA) while using the following primary antibodies: GLUT4 (Santa Cruz Biotech, CA, USA), Ser9 pGSK-3β, Thr172pAMPK, Ser473 pAkt (Cell Signaling Technology, Danvers, MA, USA), Tyr-989 pIRS-1 (Abcam, Cambridge, UK), and Thr642 pAS160 (Gene Tex, CA, USA). Histone H3 (Cell Signalling Technology, Danvers, MA, USA) served as an internal control, with the exception of pAkt, where the internal control was GAPDH (Cell Signalling Technology, Danvers, MA, USA). The bound antibodies were visualized by an ECL system (Thermo Fisher Scientific Inc., Rockford, IL, USA) and quantified using Chemi-Doc MP imaging system (Bio-Rad, Hercules, CA, USA).

### 4.10. Statistical Analysis

The results are presented as mean ± standard error of the mean (SEM). Statistical analysis was performed using SPSS v. 26.0 (SPSS Inc. Chicago, IL, USA). The analysed variables were normally distributed, according to Shapiro–Wilk´s test. Because our interest lay in determining the effectiveness of each phenolic compound and not in comparing the effects among them, comparisons between the cells treated with each compound and the control cells were made using Student’s *t* test. Statistical significance was represented, as follows: * *p* < 0.05, ** *p* < 0.01; *** *p* < 0.001

## 5. Conclusions

It can be concluded that, under our experimental conditions, neither RSV nor Q modify glucose uptake in primary myotubes, at physiological doses. Conversely, both are able to enhance glycogen synthesis and reduce lactate content, two effects that could represent a beneficial effect for glucose homeostasis and exercise endurance.

## Data Availability

Not applicable.

## Abbreviations

AK	Adenylate kinase
AKT	Protein kinase B (PKB)
AMPK	AMP-activated protein kinase
AS160	AKT Substrate of 160 kDa
ATP5a1	ATP synthase, H+ transporting, mitochondrial F1 complex, alpha subunit 1
COMT	Catechol-O-methyltransferases
COX7C	Cytochrome c oxidase subunit7C
CYCS	Cytochrome C
GLUT4	Glucose transporter 4
GSK3β	Glycogen synthase kinase 3
HK2	Hexokinase 2
IRS-1	Insulin receptor substrate 1
NDUFB8	NADH dehydrogenase (ubiquinone) 1 beta subcomplex 8
NRF1	Nuclear respiratory factor 1
PGC-1α	Peroxisome proliferator-activated receptor gamma coactivator 1-alpha
PI3K	Phosphoinositide 3-kinase
PKM	Pyruvate kinase M1/2
RSV	Resveratrol
SDHA	Succinate dehydrogenase complex, subunit alpha
SULT	Sulfotransferases
TFAM	Mitochondrial transcription factor A
UGT	Uridine-5´-diphosphate glucuronosyltransferases
Q	Quercetin
